# Trichloroethene metabolite dichloroacetyl chloride induces apoptosis and compromises phagocytosis in Kupffer Cells: Activation of inflammasome and MAPKs

**DOI:** 10.1371/journal.pone.0210200

**Published:** 2018-12-31

**Authors:** Hui Wang, Gangduo Wang, G. A. Shakeel Ansari, M. Firoze Khan

**Affiliations:** Department of Pathology, University of Texas Medical Branch, Galveston, Texas; University of Central Florida, UNITED STATES

## Abstract

Exposure to trichloroethene (TCE), an occupational and ubiquitous environmental contaminant, is associated with the development of several autoimmune diseases, including autoimmune hepatitis (AIH). However, mechanisms contributing to TCE-mediated AIH are not known. Earlier, we have shown that dichloroacetyl chloride (DCAC), one of the reactive metabolites of TCE with strong acylating capability, can elicit an autoimmune response at much lower dose than TCE in female MRL+/+ mice. Furthermore, Kupffer cells (KCs), the liver resident macrophages, are crucial for hepatic homeostasis, but can also participate in the immunopathogenesis of AIH. However, contribution of KCs in TCE-mediated AIH and the underlying mechanisms are not understood. We hypothesized that increased apoptosis and delayed clearance of apoptotic bodies, due to compromised KC function, will result in the breakdown of self-tolerance, autoimmunity, and ultimately AIH. Therefore, using an *in vitro* model of immortalized mouse KCs, we investigated the contribution of DCAC in TCE-mediated AIH. KCs were treated with different concentrations of DCAC and apoptosis was measured by Annexin V and PI staining. Also, the impact of DCAC on phagocytic potential of KCs was evaluated. Furthermore, markers of inflammasome (NLRP3 and caspase1) were analyzed by real-time PCR and Western blot analysis. DCAC treatment resulted in significantly increased early and late-stage apoptosis, accompanied with inflammasome activation (NLRP3 increases). DCAC treatment resulted in decreased phagocytic function of KCs in a dose-dependent manner, with reduced MFG-E8 levels (phagocytotic function). Furthermore, DCAC exposure led to induction of phos-ERK and phos-AKT signaling. These findings suggest that DCAC induces apoptosis and inflammasome activation, while compromising the phagocytic function of KCs. Our data support that increased apoptosis and impaired KC function by DCAC could be contributory to TCE-mediated AIH.

## Introduction

Trichloroethene (trichloroethylene, TCE) is a chlorinated toxic solvent and widely used as industrial degreaser. Exposure to TCE is associated with several autoimmune diseases, including autoimmune hepatitis (AIH) [1–3]. AIH is a global disease in all ages and ethnicities with a female predominance [4]. AIH is characterized by loss of tolerance against self-antigens, where autoreactive T cells lead to progressive liver injury and ultimately failure [5]. However, the precise aetiology of AIH is not fully understood.

It has been shown that TCE exerts its toxic effects primarily in liver and kidney through its metabolites [6]. Earlier, we have demonstrated that exposure to dichloroacetyl chloride (DCAC), one of the metabolites of TCE with strong acylating property, causes induction of autoimmune response, evident from several increased autoantibodies [1, 7, 8]. Kupffer cells (KCs) are key component of hepatic innate immune system through their role in phagocytosis, antigen presentation and cytokine production [9]. KC-mediated phagocytosis is critical for the clearance of the pathogens, antigens or apoptotic bodies to maintain the homeostasis [9]. Compromised phagocytic function of KCs may then cause the accumulation of apoptotic cells/bodies, generation of neoantigens, leading to initiation of inflammatory responses in the liver and subsequently AIH [10]. The dysregulation of immune response in AIH is initiated by presentation of self-antigens to naïve T cells by antigen presentation cells, like dendritic cells (DCs) and KCs [11, 12]. Naïve CD4 T cells then differentiate into Th1, Th2 and Th17 cells, which are associated with the secretion of different cytokines, contributing to the pathogenesis of AIH [13, 14]. Numerous studies have demonstrated that IL17 levels correlate with the hepatic inflammation and fibrosis in patients with AIH [13, 15]. Furthermore, our previous study has shown that long-term TCE exposure (24 or 36 weeks) results in decreased number and function of KCs in the livers of female MRL+/+ mice [2], suggesting that KC function is critical in the pathogenesis of TCE-mediated AIH. However, contribution of DCAC in TCE-mediated KC dysfunction, cytotoxicity and/or AIH and underlying mechanisms are largely unknown.

TCE-mediated AIH is associated with increased oxidative stress, cytokine production and apoptosis in the liver [16, 17]. NOD-like receptor protein 3 (NLRP3) inflammasome is activated by ROS production, resulting in inflammation and programmed cell death in liver disorders, such as viral hepatitis, non-alcoholic fatty liver disease and AIH [18–21]. Activation of NLRP3 may be an important mechanism by which TCE or its metabolites induce an immune response that leads to AIH in MLR+/+ mice. Therefore, to understand the mechanism of TCE-mediated AIH, the present study examined the potential of DCAC, a reactive metabolite of TCE, in inducing inflammasome activation, apoptosis, and impaired phagocytosis of KCs *in vitro* using murine kupffer cells.

## Materials and methods

### Reagents

Primary antibodies against AKT, phosphor-AKT (S473), phosphor-AKT (T308), phosphor-ERK, phosphor-mTOR (Ser2448), ERK and NLRP3 were purchased from Cell Signaling Technology (Danvers, MA). Antibodies against MFG-E8 and Caspase1 were purchased from R&D systems (Minneapolis, MN). Immortalized murine kupffer cell line was obtained from EMD Millipore [22], and DCAC was obtained from Sigma-Aldrich (St. Louis, MO). FluoSpheres Carboxylate-Modified Microspheres, size 1.0 μm were purchased from Thermo Fisher Scientific (Waltham, MA).

### Apoptosis assays

Kupffer cells (KCs) were cultured with complete RPMI 1640 medium and were used for experiments before passage 5. KCs were plated in 12-well plates overnight and then treated with DCAC (0, 1, 2.5 or 5 mM) for 24 h. Cells were detached using accutase cell detachment solution (BD, Franklin Lakes, NJ), followed by APC-labeled Annexin-V and PI staining. Data were acquired by BD LSR Fortessa, and were analyzed by Flowjo10 software (Franklin Lakes, NJ).

### Phagocytosis assays

For phagocytosis analysis, KCs were treated with DCAC as described above. After 24 h, FluoSpheres Carboxylate-Modified Microspheres (200 particles/cell) were added and incubated for 15 min. The supernatant was decanted and the cells were washed with PBS for five times. Fluorescent images were taken from 10 different fields per treatment. The FluoSphere positive cells and the total cells per image were counted to calculate the ratio of FluoSphere positive cells to total cells.

### Real time PCR

Total RNA was extracted from cells using RNeasy Mini Kit (Qiagen, Valencia, CA). cDNA was synthesized with iScript^™^ cDNA synthesis kit (Bio-Rad Laboratories, Hercules, CA). Real-time PCR was conducted according to Wang et al [17]. The mRNA expression of selected genes related to inflammation, inflammasome and phagocytosis were analyzed. Mouse glyceraldehyde 3-phosphate dehydrogenase (GAPDH) was used as the housekeeping gene. Primer sequences are listed in S1 Table.

### Western blot analysis

Protein samples from lysed cells were prepared in RIPA buffer (Cell Signaling Technology, Danvers, MA) supplemented with protease inhibitors (Sigma-Aldrich, St. Louis, MO). Ten μg of protein lysates were subjected to Western blot analyses using antibodies against specific target proteins. Immunoblot images were quantified using Image Studio Lite Ver 5.2 and the intensity was determined and normalized to a respective loading control protein GAPDH.

### Statistical analysis

All values are expressed as means ± SEM. Intergroup differences were appropriately assessed by one-way analysis of variance (ANOVA) followed by Tukey-Kramer multiple comparisons test. The p values <0.05 were considered to be statistically significant. * p < 0.05 and ** p < 0.01.

## Results

### DCAC-induced early and late stage apoptosis in KCs

**Fig 1:**
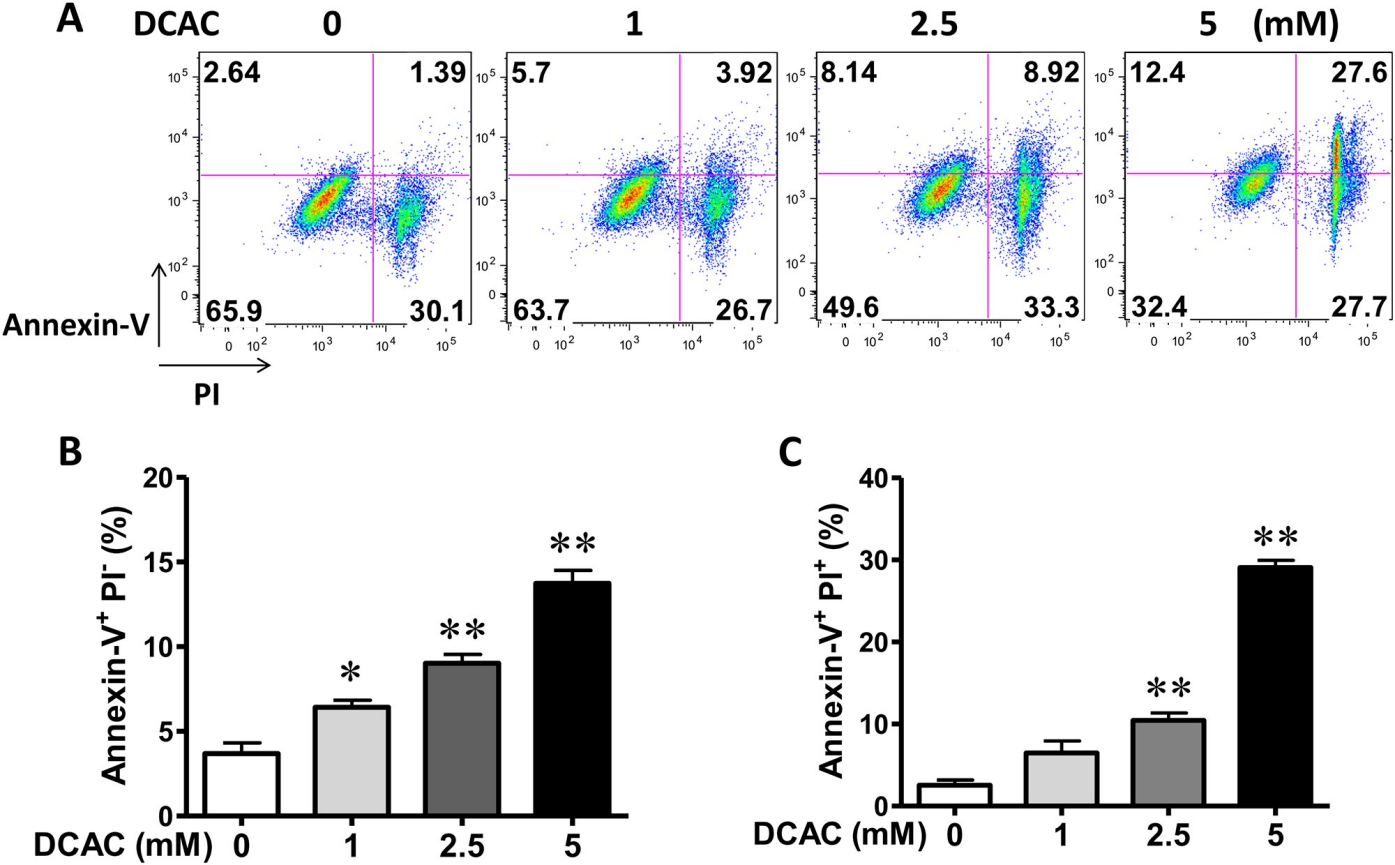
Effect of DCAC on apoptosis of KCs. Cells were exposed to DCAC at concentrations of 0, 1, 2.5 or 5 mM for 24 h. Cell apoptosis was determined by Annex V staining and detected by FACS. (A) Flow cytometry analysis of cell apoptosis. (B) The percentage of early and late-stage apoptotic cells are represented in histogram. n = 3 in each group. *p<0.05; **p<0.01, as compared to the control.

Previous studies have shown that TCE induces autoimmune responses in MLR+/+ mice [1]. To evaluate the contribution of DCAC in TCE-mediated autoimmunity, we investigated the effect of DCAC exposure on cell apoptosis *in vitro*. Early apoptosis and late apoptosis were quantified using annexin V and propidium iodide staining by FACS, and quantified as Annexin V^+^ PI^-^ and Annexin V^+^ PI^+^ cells (Fig 1A). DCAC treatment resulted in dose-dependent and statistically significant increases in both early- and late-stage apoptosis after 24 h exposure, with higher percentage of Annexin V^+^PI^-^ and Annexin V^+^ PI^+^ cells (Fig 1B and 1C).

### DCAC inhibited the phagocytic activity of KCs

**Fig 2:**
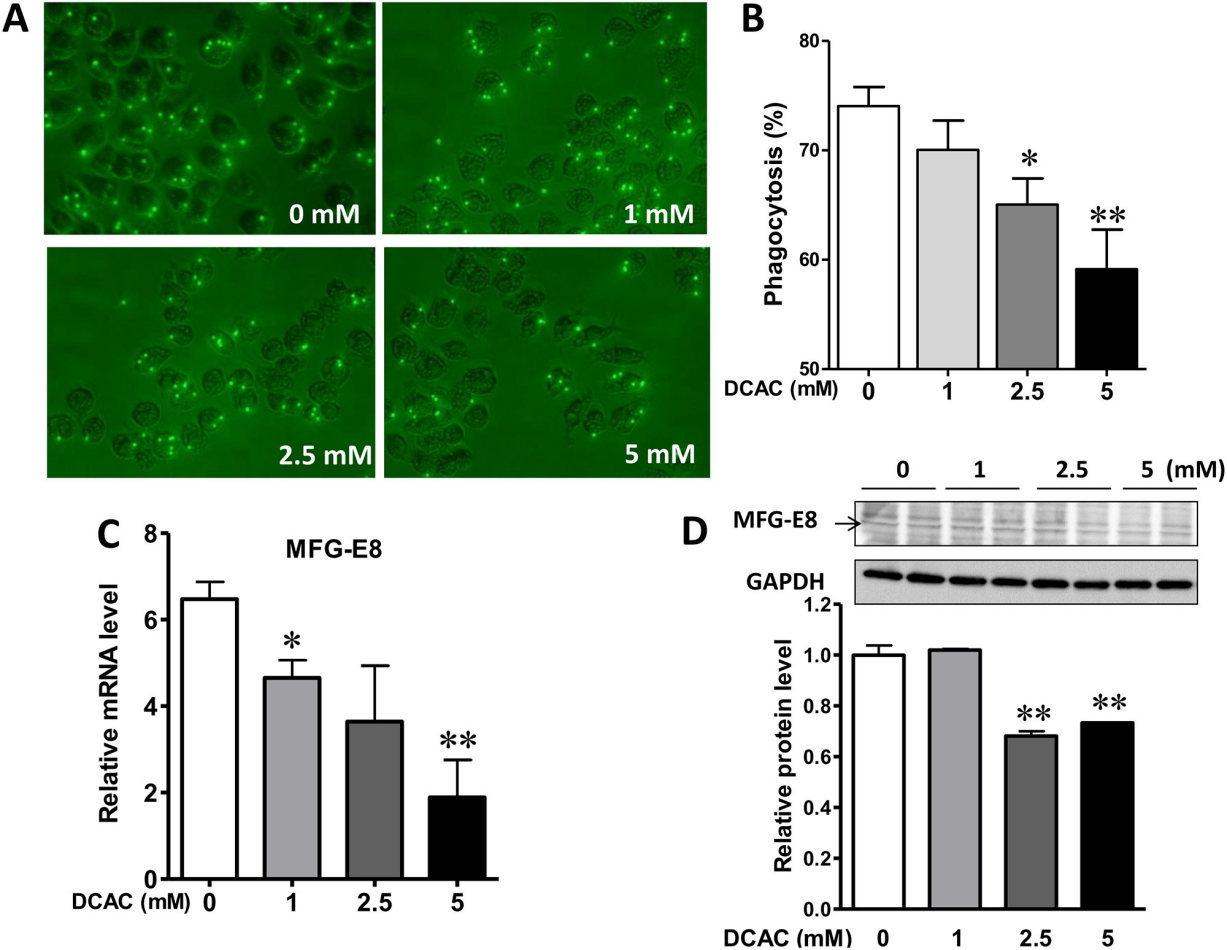
Effect of DCAC on KC phagocytosis. Cells were treated with different concentrations of DCAC for 24 h, and then incubated with fluorescent latex beads for 30 min. (A) Representative images of KCs treated with DCAC (0, 1, 2.5 or 5 mM) for 24 h, showing decreased phagocytic activity after DCAC exposure. (B) The KC phagocytosis was measured by counting the bead-internalized KCs from 10 different fields and the percentage of bead-positive cells was calculated, (C) Relative mRNA level of MFG-E8 (phagocytic function), as measured by RT-PCR, and (D) Relative protein level of MFG-E8, as determined by Western blotting. n = 3 in each group. *p<0.05; **p<0.01, as compared to the control.

One of the major roles of KCs is to clear the apoptotic bodies from the injured liver, and delayed clearance due to compromised phagocytic function of KCs could lead to activation of hepatic inflammatory responses, eventually eliciting an autoimmune response [23]. Therefore, we examined the effect of DCAC on the phagocytic activity and determined the effect of DCAC on KC function. DCDC exposure led to decreased phagocytic activity, evidenced by reduced number of cells positive for beads (Fig 2A and 2B). Moreover, the number of beads per cell also tended to decrease after DCAC treatment. Milk factor globule EGF factor 8 (MFG-E8), which represents a link between apoptotic cells and phagocytes, enhances clearance of apoptotic cells and suppresses inflammatory responses [24]. We, therefore, also investigated the alterations in MFG-E8 expression in KCs after DCAC exposure. MFG-E8 expression was markedly reduced after DCAC exposure (Fig 2C and 2D). These results indicate that DCAC exposure down-regulates MFG-E8 expression and reduces phagocytic activity.

### DCAC modulated iNOS expression and inflammatory cytokines

**Fig 3:**
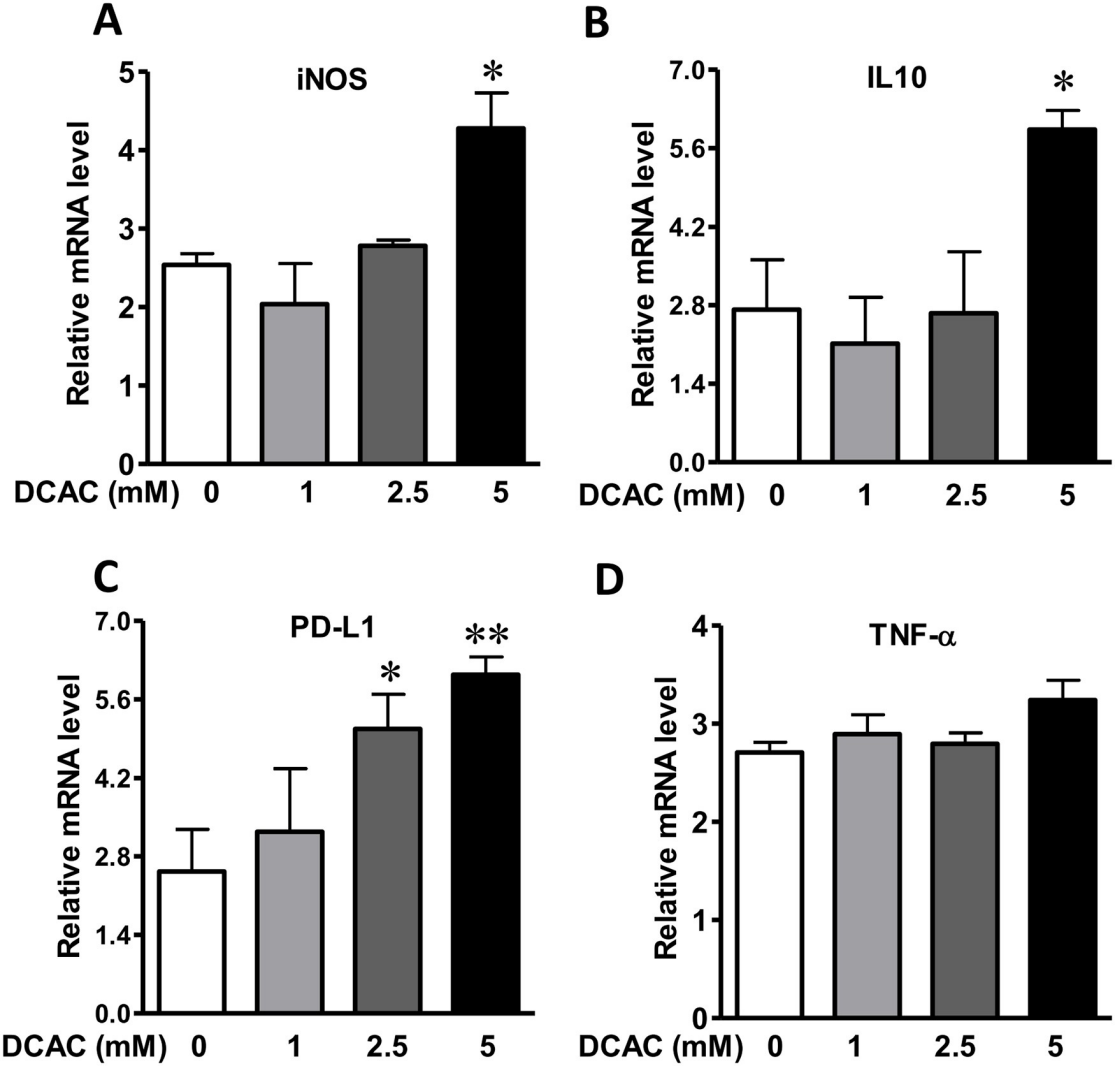
Effect of DCAC on the expression of iNOS, inflammatory cytokines and apoptosis related gene in KCs. KCs are pre-treated with DCAC for 24 h and cultured with or without LPS (200 ng/ml) for 4 h. qPCR was performed to determine the expression of iNOS and inflammatory cytokines: iNOS (A), IL-10 (B), TNF-α (C) and PD-L1 (D). n = 3 in each group. *p<0.05 vs. the controls.

**Fig 4:**
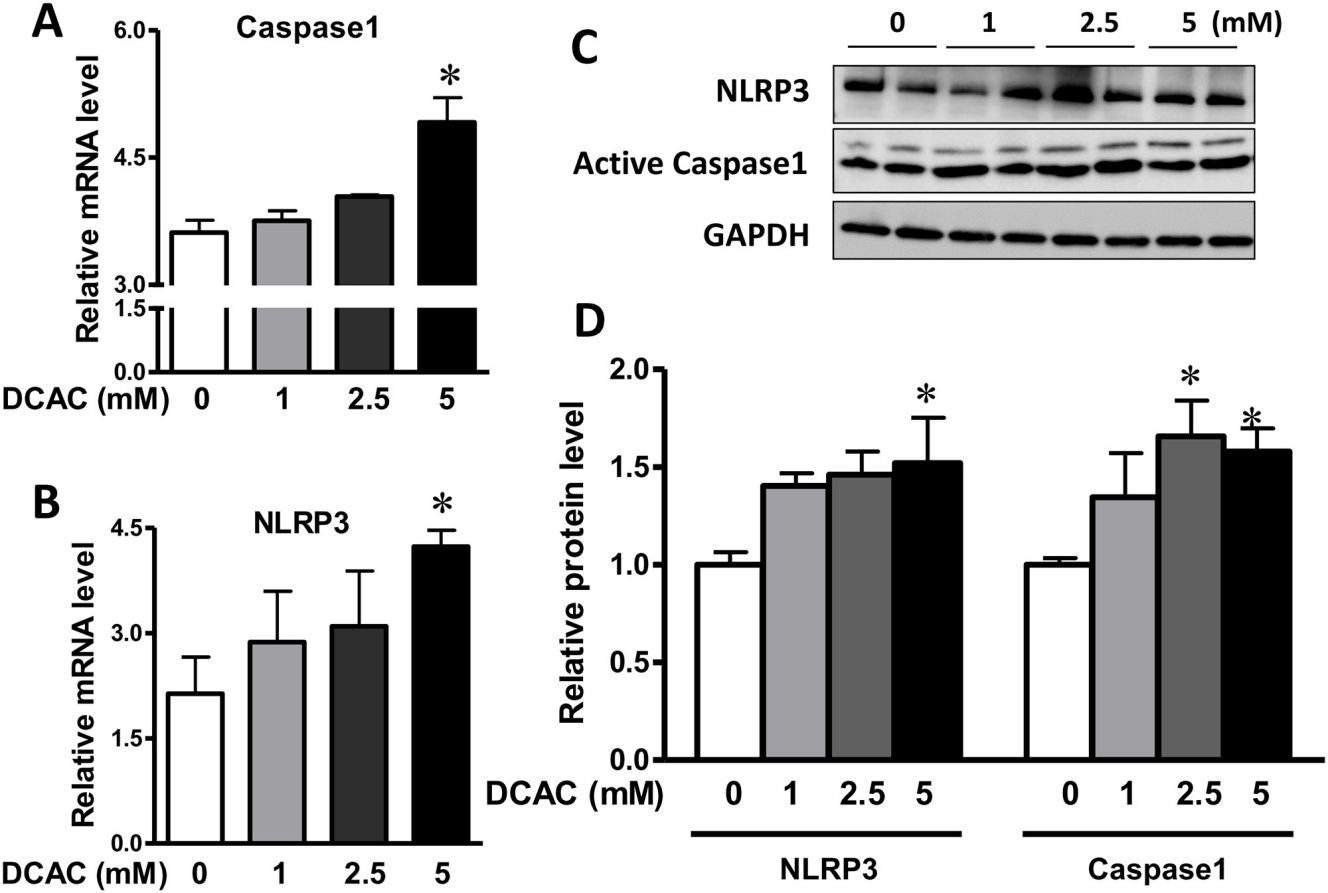
DCAC promoted inflammasome activation in KCs. KCs were pre-treated with DCAC at concentrations of 0, 1, 2.5 or 5 mM for 24 h. qPCR and Western blot analyses were done for the expression of inflammasome (NLRP3 and caspase1). (A, B) mRNA levels of the caspase1 and NLRP3, as measured by RT-PCR, and (C) Relative protein levels of NLRP3 and caspase1, as determined by Western blotting. n = 3 in each group.*p<0.05 vs. the controls.

Activated KCs secrete cytokines and chemokines to recruit and activate innate immune cells, which could play a pivotal role in the pathogenesis of AIH [25]. To assess whether DCAC treatment can elicit an inflammatory response in KCs, DCAC pre-treated KCs were stimulated with LPS (100 ng/ml) for 4h. Elevated expression of iNOS and IL10 was observed in KCs treated with higher concentration of DCAC (Fig 3A and 3B), which was also associated with increased caspase1 and NLRP3 expression (Fig 4A and 4B). Moreover, up-regulation of PD-L1 was also observed following DCAC treatment at 2.5 and 5 mM (Fig 3C). However, no significant difference was observed in TNF-α level following DCAC exposure (Fig 3D).

### DCAC induced inflammasome activation

Inflammasome activation in KCs results in a wide range of immune responses, including cytokine production, immune cell differentiation and programed cell death [20, 26, 27]. We determined the effect of DCAC on inflammasome activity to evaluate whether DCAC-induced apoptosis also involved inflammasome activation. Activated caspase1 and NLRP3 were measured at both mRNA and protein levels after DCAC treatment. Activated caspase 1 was significantly increased with DCAC treatment at 2.5 and 5 mM (Fig 4A and 4C). More importantly, both mRNA and protein levels of NLPR3 showed increasing trends and were significantly higher following exposure to 5 mM DCAC (Fig 4B and 4C).

### DCAC up-regulates AKT-mTOR and MAPK signaling pathways

**Fig 5:**
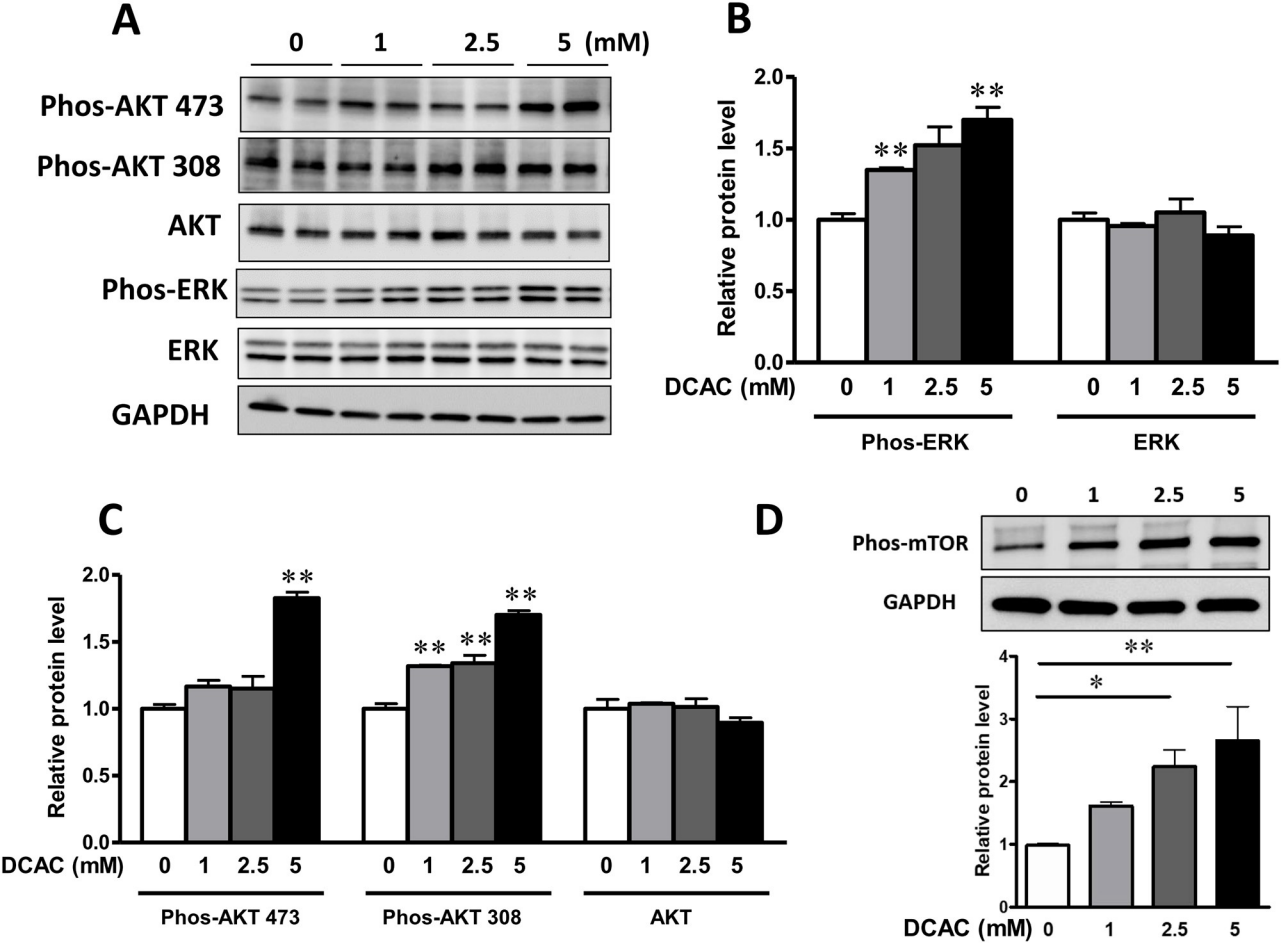
DCAC up-regulated the AKT/mTOR and MAPK signaling in KCs. KCs were pre-treated with DCAC at concentrations of 0, 1, 2.5 or 5 mM for 24 h. (A) Representative images of the cellular levels of phos-AKT S473, phos-AKT T308, total AKT, phos-ERK and ERK, as determined by Western blotting, (B) Relative phos-ERK level, as determined by Western blotting, (C) Relative protein levels of phos-AKT(Ser-473) and phos-AKT(Thr-308), as determined by Western blotting, and (D) Representative images and relative phos-mTOR, as determined by Western blotting. n = 3 in each group. *p<0.05; **p<0.01, as compared to the controls.

To further investigate the mechanism of DCAC-induced apoptosis and compromised phagocytic KC function, we evaluated the effects of DCAC on reactive oxygen species (ROS) sensitive AKT and ERK phosphorylation. AKT-mTOR activation has been reported as a pro-death signal in insulin-induced programed cell death [28]. In KCs, phosphorylation of phos-AKT Ser-473, phos-AKT Thr-308 and downstream mTOR were significantly increased in response to DCAC treatment (Fig 5A, 5C and 5D). Furthermore, DCAC exposure exhibited significantly increased levels of phospho-ERK (Fig 5A and 5B) among the MAPKs.

## Discussion

TCE is an extensively used industrial solvent and a ubiquitous environmental contaminant. TCE exposure has been associated with autoimmune responses in both humans and experimental animals [1, 3, 29]. Long-term exposure to TCE via drinking water has been shown to result in the induction of AIH in MRL+/+ mice, evidenced by increased apoptosis and reduced number and function of KCs [2]. TCE-mediated AIH is also associated with increased hepatic inflammation in MRL+/+ mice [30]. However, mechanism(s) by which TCE leads to induction of AIH is not well understood. Interestingly, DCAC, one of TCE metabolites with strong acylating capacity, induces an autoimmune response in MRL+/+ mice at a much lower concentration than TCE [7], indicating that DCAC formation may be a contributory factor in TCE-induced AIH. In this study, we aimed to further evaluate the contribution of DCAC in TCE-mediated AIH by examining early molecular events in KCs following DCAC treatment *in vitro*. Our results in this study show that DCAC induces both early and late-stage apoptosis in KCs, inhibits their phagocytic potential, and activates inflammasome as well as MAPK signaling.

Apoptosis plays an important role in various liver diseases [31]. Previous studies have shown that TCE exposure leads to alteration/induction of apoptosis-related genes in hepatocytes [32]. Our data in this study show that DCAC could be an inducer of both early and late-stage apoptosis in KCs. These findings are consistent with previous results and strongly support that TCE exposure induces AIH through increased hepatic apoptosis, which was also evidenced by greater TUNEL staining [2]. Apoptosis is the initial event which could result in non-specific inflammation due to ineffective clearance of the apoptotic bodies by functionally compromised KCs [12, 31, 33]. Our results thus suggest that DCAC-mediated apoptosis in KCs may be a mechanism contributing to TCE-mediated AIH *in vivo*.

KCs/macrophages are key components of the innate immune system which via phagocytosis, destroy and clear the dead bodies, foreign materials and microbes [34, 35]. KCs are also involved in the immune-mediated liver diseases, and their phagocytic activity is reported to be impaired in TCE-induced hepatitis [2]. The phagocytic activity of peripheral blood mononuclear cells is also significantly impaired in patients with AIH [10]. To determine the direct impact of DCAC treatment on the function of KCs, we evaluated KC’s phagocytic activity and a marker protein (MFG-E8) related to its function. Our data showed that DCAC exposure reduced the phagocytic function of KCs, as evident from inhibition in the phagocytosis of fluorescent latex beads. MFG-E8 is a phosphatidylserine recognition protein, enhancing the phagocytosis of apoptotic cells [36]. MFG-E8 exhibits protective role via attenuating inflammatory responses in systemic lupus erythematosus [37] and reducing ROS in subarachnoid hemorrhage [38]. MFG-E8 deficiency promotes autoimmunity by delaying clearance of dying cells and altering intracellular antigen processing, leading to autoimmune diseases [39]. Our results showed that the expression of MFG-E8 was significantly decreased after DCAC exposure, suggesting that DCAC impaired the phagocytic function of KC via down-regulating MFG-E8, and this could potentially contribute to TCE-mediated AIH.

**Fig 6:**
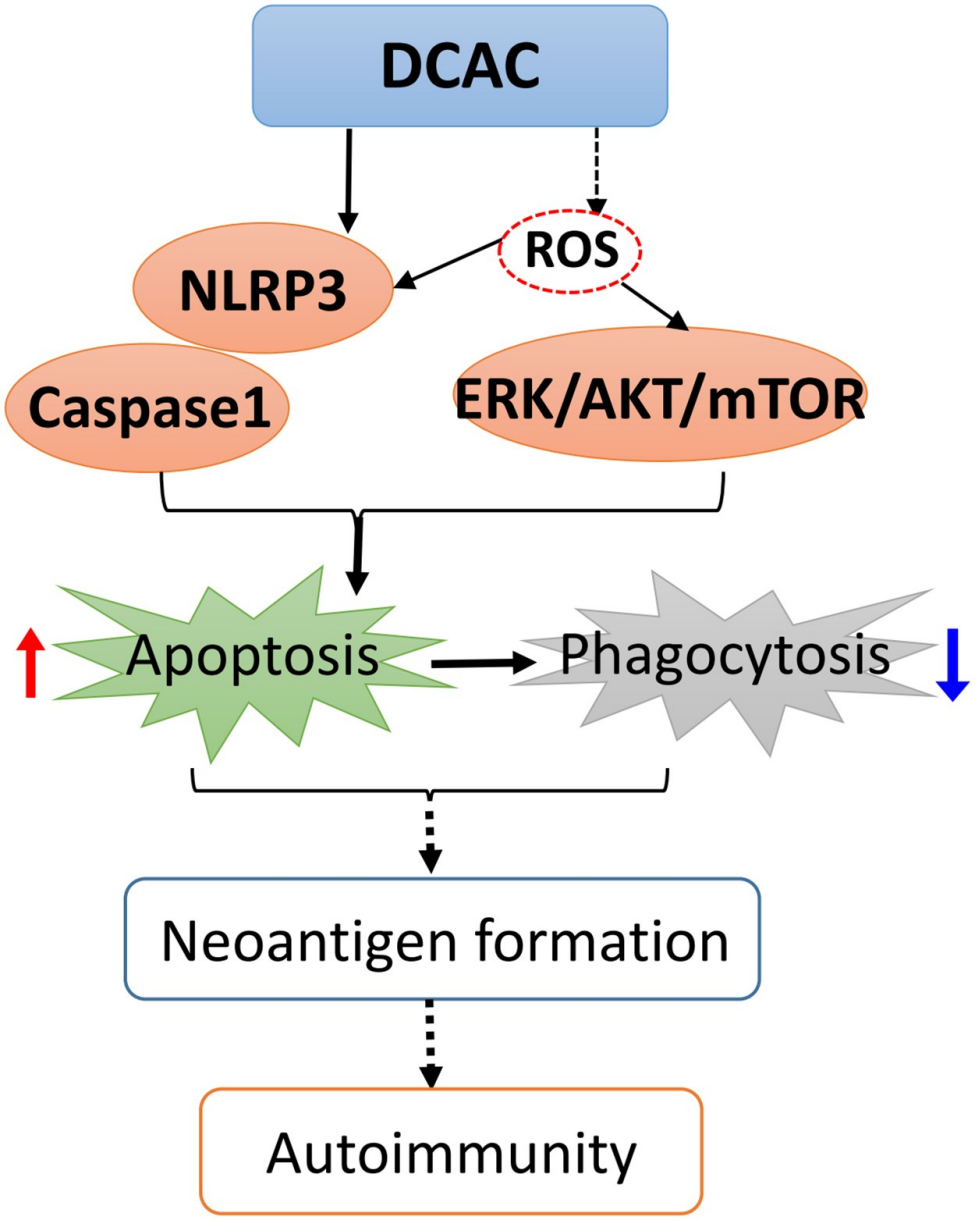
The plausible mechanism of DCAC-induced autoimmune response. DCAC can induce inflammasome NLRP3/caspase1 and ROS-related ERK/AKT/mTOR activation in Kupffer cells, leading to increased apoptosis and impaired phagocytosis. Ultimately, delayed clearance of apoptotic bodies due to compromised Kupffer cell function will result in the breakdown of self-tolerance, autoimmunity, and eventually AIH. “------”, established with parent compound TCE.

To determine the signaling pathways involved in DCAC-induced apoptosis and impaired phagocytosis, we evaluated the apoptosis related inflammasome activation. Our data show a significant upregulation of NLRP3, a novel regulator of inflammation and cell death, along with active caspase1 after KCs were exposed to DCAC, suggesting the involvement of inflammasome activation during DCAC-induced apoptosis. NLRP3 activation has been reported to induce pyroptosis and apoptosis, and NLRP3-dependent IL-1β production could contribute to the pathogenesis of AIH [40]. Our laboratory previously reported that TCE exposure causes oxidative stress (OS) and also iNOS induction (nitrosative stress) [17, 41, 42]. Furthermore, iNOS knockout MRL+/+ mice show attenuation of TCE-induced autoimmunity [43]. In the present study, the observed increase in iNOS level after DCAC treatment, suggests that DCAC is not only a strong acylating metabolite, but also ROS/reactive nitrogen species (RNS) inducing agent. PD-L1 level in macrophages has been correlated with nitric oxide production and serves as a marker for “primed” inflammatory macrophages [44]. In AIH patients, PD-L1 was co-localized with macrophages and its mRNA expression in the liver was significantly increased [45]. Elevated PD-L1 level was also observed in KCs after DCAC treatment in this study, which is consistent with increased NLRP3 and caspase1 levels. Since increasing evidences provide a link between ROS generation and activation of MAPK signaling pathways [46–48], we, therefore, examined if DCAC also modulated MAPK signaling in KCs. In fact, we observed a dose-dependent induction of phos-ERK, a member of the MAP kinase family of serine/threonine kinases. Our findings are thus consistent with earlier observations that ERK activation could promote intrinsic and/or extrinsic apoptosis in various cell types including KCs [49, 50]. AKT is a metabolic regulator in macrophages activated by intracellular ROS [51, 52], and AKT activation is associated with inflammation and IL-10 production in macrophages [53, 54]. Here, we observed that DCAC exposure increased the expression of phos-AKT Ser-473 and phos-AKT Thr-308, as well as its downstream phos-mTOR. Our findings on DCAC-induced increases in phos-ERK and phos-AKT/mTOR are consistent with elevated ROS/RNS observed in our previous *in vivo* studies and provide further support to the role OS in TCE-mediated autoimmunity [17, 43]. The key findings on DCAC-induced inflammasome activation, apoptosis and compromised phagocytosis of KCs, leading to Neoantigen formation, and ultimately to AIH are presented in Fig 6.

In summary, our study showed that TCE metabolite DCAC led to increased apoptosis and impaired phagocytic function of KCs. Additionally, we demonstrated that DCAC induced the activation of inflammasome, phos-ERK and phos-AKT/mTOR. Our data thus suggest that increased apoptosis and impaired KC phagocytic function by DCAC could be an important mechanism by which TCE could mediate AIH.

## Supporting information

S1 Table Real-time PCR primers for qRT-PCR assays. (DOCX)
