# Supplementary material for: Trichloroethene metabolite dichloroacetyl chloride induces apoptosis and compromises phagocytosis in Kupffer Cells: Activation of inflammasome and MAPKs
Source: PLoS One. 2018 Dec 31;13(12):e0210200. doi: 10.1371/journal.pone.0210200 (PMC6312261; doi:10.1371/journal.pone.0210200)
Supplement: S1 Table — (DOCX) [file pone.0210200.s001.docx]

**Table S1. Real-time PCR primers for qRT-PCR assays**

**Gene Name Primer Sequence**

GAPDH Forward 5’-TGGAAAGCTGTGGCGTGAT-3’

Reverse 5’-TGCTTCACCACCTTCTTGAT-3’

TNF-α Forward 5’-CCCTCACACTCAGATCATCTTCT-3’

Reverse 5’-CTTTGAGATCCATGCCGTTG-3’

IL-10 Forward 5’- GCTCTTACTGACTGGCATGAG -3’

Reverse 5’- CGCAGCTCTAGGAGCATGTG -3’

iNOS Forward 5’- GTTCTCAGCCCAACAATACAAGA -3’

Reverse 5’- GTGGACGGGTCG ATGTCAC -3’

NLRP3 Forward 5’- ATGCTGCTTCGACATCTCCT -3’

Reverse 5’- AACCAATGCGAGATCCTGAC -3’

PD-L1 Forward 5’- GCTCCACCGGACTTGTACGTG -3’

Reverse 5’- TGATCTGAAGGGCAGCATTTC -3’

Caspase 1 Forward 5’- AGATGGCACATTTCCAGGAC -3’

Reverse 5’- GATCCTCCAGCAGCAACTTC -3’

MFG-E8 Forward 5’- CAGCAACTATGATAGCAAGCCC -3’

Reverse 5’- CCTGCGTCATCACACCTGATA -3’
